# Oral and dental late effects in long-term survivors of childhood embryonal brain tumors

**DOI:** 10.1007/s00520-022-07405-8

**Published:** 2022-10-29

**Authors:** Kristine Eidal Tanem, Einar Stensvold, Petter Wilberg, Anne B. Skaare, Petter Brandal, Bente Brokstad Herlofson

**Affiliations:** 1 Department of Oral Surgery and Oral Medicine, Faculty of Dentistry, University of Oslo, Oslo, Norway; 2 Department of Pediatrics, Oslo University Hospital, Oslo, Norway; 3 The Faculty of Medicine, Institute of Clinical Medicine, University of Oslo, Oslo, Norway; 4 Oral Health Centre of Expertise in Eastern Norway (OHCE), Oslo, Norway; 5 Department of Pediatric Dentistry and Behavioral Science, Faculty of Dentistry, University of Oslo, Oslo, Norway; 6 Division of Cancer Medicine, Dep. of Oncology, Oslo University Hospital, Oslo, Norway; 7 Section for Cancer Cytogenetics, Institute for Cancer Genetics and Informatics, Oslo University Hospital, Oslo, Norway; 8 Division for Head, Neck, and Reconstructive Surgery, Unit of Oral and Maxillofacial Surgery, Department of Otorhinolaryngology, Oslo University Hospital, Oslo, Norway

**Keywords:** Pediatric brain tumors, Survivors, Late effects, Oral and dental, Tooth abnormalities, Dental caries

## Abstract

**Purpose:**

To investigate oral and dental late effects in survivors of childhood brain tumors medulloblastoma (MB) and central nervous system supratentorial primitive neuroectodermal tumor (CNS-PNET).

**Methods:**

This cross-sectional study assessed oral and dental late effects in MB/CNS-PNET survivors treated before 20 years of age, and with a minimum of 2 years since treatment. Participants went through an oral and radiographic examination. We assessed oral status using the decayed-missing-filled index (DMFT), oral dryness, maximum mouth opening (MMO), fungal infection, and registration of dental developmental disturbances (DDD) in the form of hypodontia, microdontia, and enamel hypoplasia.

**Results:**

The 46 participants’ mean age at enrolment was 27 ± 12.8 years and at treatment 8.5 ± 5.2 years, and the mean time since treatment was 18.9 ± 12 years. Over a third (35%) of survivors had reduced mouth opening (mean 29.3 ± 5.6 mm (range 16–35)). A significantly lower MMO was found in individuals treated ≤ 5 years compared to survivors treated > 5 years (*p* = 0.021). One or more DDD were registered in 30.4% of the survivors, with a significantly higher prevalence in individuals treated ≤ 5 years (*p* < 0.001). Hypodontia was the most prevalent type of DDD. There was no difference in DMFT score in relation to age at treatment. Oral dryness was not frequently reported or observed in these survivors.

**Conclusion:**

Survivors of childhood MB/CNS-PNET are at risk of oral and dental late effects including reduced mouth opening and DDD. The risk is highest in survivors treated before the age of 5.

## Introduction

Medulloblastoma (MB) and central nervous system supratentorial primitive neuroectodermal tumor (CNS-PNET) are malignant brain tumors [1] accounting for 15–20% and 2.5% of all pediatric brain tumors respectively [2, 3]. Although survival rates have improved in the last decades [3], the multimodal treatment including surgery, chemotherapy, and/or radiotherapy (RT) [2, 4] entails a high risk of severe late effects [3–5]. RT to the CNS axis is necessary for cure and is administered to all patients except individuals under the age of 3–5 years who are, despite a consequently lower cure potential, most often treated without RT due to its high risk of late neurocognitive impairment [2, 4]. Chemotherapy often consists of combinations of different chemotherapeutic agents including vincristine and alkylating agents such as cyclophosphamide [2].

Survivors of childhood cancer are at risk of oral and dental late effects [6, 7]. Young age at treatment and certain treatment modalities seem to increase prevalence and/or severity of these late effects [6–8]. Treatment before the age of 5 years is associated with a higher risk of dental developmental disturbances (DDD) than treatment at a later age [8, 9]. Additionally, the risk of DDD may increase further if the treatment includes cranial irradiation [10] and/or exposure to high doses of chemotherapeutic agents [9, 11]. Dental caries is reported to be higher in survivors of childhood cancers compared to healthy age- and gender-matched controls [12, 13]. The risk of dental caries may be related to age at treatment, as its prevalence is reported to be higher in patients treated after the age of 5 years compared to patients treated before 5 years of age [9, 13]. Pediatric cancer survivors treated with high doses of the chemotherapeutic drug cyclophosphamide may experience reduced salivary flow [11]. Additionally, patients receiving craniospinal irradiation (CSI) are at risk of developing xerostomia and secondary malignancies in the parotid glands [14].

Although there are several studies on oral and dental late effects in survivors of childhood cancer treatment [8–12, 15–17], only a few studies included small numbers of pediatric brain tumor survivors in their study population [14, 18–20]. No studies have assessed oral and dental late effects in a homogenous group of MB/CNS-PNET survivors. Considering the risk of irreversible treatment-related damage to oral tissues, more knowledge on the type and frequency of oral late effects is needed. Thus, the aim of this study was to investigate oral and dental late effects in long-term survivors of childhood MB/CNS-PNET.

## Methods

### Study design and participants

**Fig. 1:**
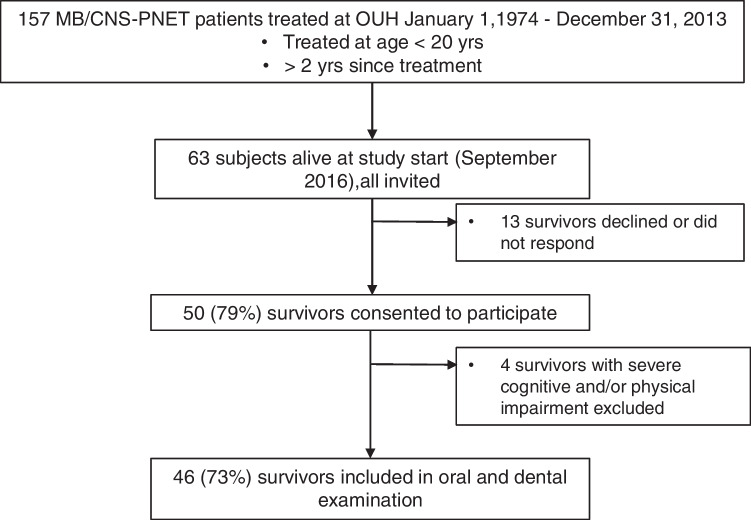
Flowchart illustrating recruitment of study population. Abbreviations: MB medulloblastoma, CNS-PNET central nervous system supratentorial primitive neuroectodermal tumor, OUH Oslo University Hospital

This cross-sectional descriptive study of oral and dental late effects was a sub-study of a large multidisciplinary study at Oslo University Hospital (OUH) [2, 5, 21, 22]. Survivors of pediatric MB/CNS-PNET were identified using the archives of Department of Pathology, surgical protocols at Department of Neurosurgery, and data from the Cancer Registry of Norway [5]. To be included in the study, the participants had to (1) be treated ≤ the age of 20 years at OUH between January 1, 1974, and December 31, 2013, (2) have a histophatologically confirmed MB/CNS-PNET, and (3) have a minimum of 2 years since treatment. During the selected study period, 157 patients treated for MB/CNS-PNET were identified, of which 63 subjects were alive, and invited to participate. Recruitment of participants is described in Fig. 1.

An oral examination was performed by a dentist in a dental office at OUH between September 2016 and October 2018. Clinical photographs of the oral cavity including oral mucosa and teeth were taken from all survivors. In addition, panoramic radiographs were taken with Planmeca PM 2002 CC Proøine Pan/Ceph (Planmeca Oy, Helsinki, Finland). Survivors who could not complete oral and/or radiographical examination due to severe neurocognitive and/or physical disabilities were excluded from the study.

### Oral health parameters

#### Dental caries

Dental caries was evaluated in each participant using the decayed-missing-filled teeth index for permanent teeth (DMFT), or for deciduous teeth (dmft), according to the World Health Organization criteria [23]. The total DMFT and dmft scores ranged from 0 to 28 and from 0 to 20, respectively. Third molars were excluded.

#### Oral dryness

To assess patient-reported oral dryness, the participants were asked to fill in the Norwegian translation of the Summated Xerostomia Inventory-Dutch Version (SXI-D) questionnaire [24]. This SXI-D is a validated patient-reported questionnaire and consists of five statements: (1) my mouth feels dry when eating a meal, (2) my mouth feels dry, (3) I have difficulty eating dry food, (4) I have difficulties swallowing dry food, and (5) my lips feel dry. Participants were asked to choose from three possible response categories: “never” (score 1), “occasionally” (score 2), or “often” (score 3), with a summated score ranging from 5 to 15, where a higher score indicated a severe problem related to oral dryness [24].

Observer-rated oral dryness was assessed using the Clinical Oral Dryness Score (CODS) index [25] and the dental mirror friction test; the latter is a screening method for saliva lubrication of mucous membranes [26]. CODS index is a grading measure consisting of 10 features of oral dryness; each positive feature scores 1 point [25]. A total score was given for each participant: 1–3 mild dryness, 4–6 moderate dryness, and 7–10 severe dryness [25, 27]. The dental mirror friction test was performed by sliding a dental mirror along the buccal mucosa, and the presence of friction was assessed as follows: 0 = no friction and 1 = friction [26].

#### Maximum mouth opening (MMO) measurement

MMO was measured with a metallic ruler from the incisal edge of upper frontal teeth to incisal edge of lower frontal teeth, three times in each participant. The mean score was calculated, and MMO ≤ 35 mm was used as a cut-off value for reduced mouth opening [28].

#### Oral fungal infection

The presence of oral fungal infection was assessed by clinical examination supplemented by a microbiological test. Separate sterile cotton swabs were rubbed against two oral sites: (1) the anterior part of the tongue and (2) the buccal mucosa. Swab samples were inoculated onto CHROMagar (CHROMagar™ Candida, Paris/France) culture plates and incubated at 37 °C for 48 h. A diagnosis of oral candidiasis was made only in cases where clinical as well as microbiological findings were positive [29].

### Dental developmental disturbances

DDD were assessed by a clinical and radiographic examination. Since age at diagnosis and treatment may impact DDD [8, 9], the survivors were separated in 2 age groups when DDD were addressed: (1) survivors treated ≤ 5 years of age, (2) survivors treated > 5 years of age. Third molars were excluded.

**Fig. 2:**
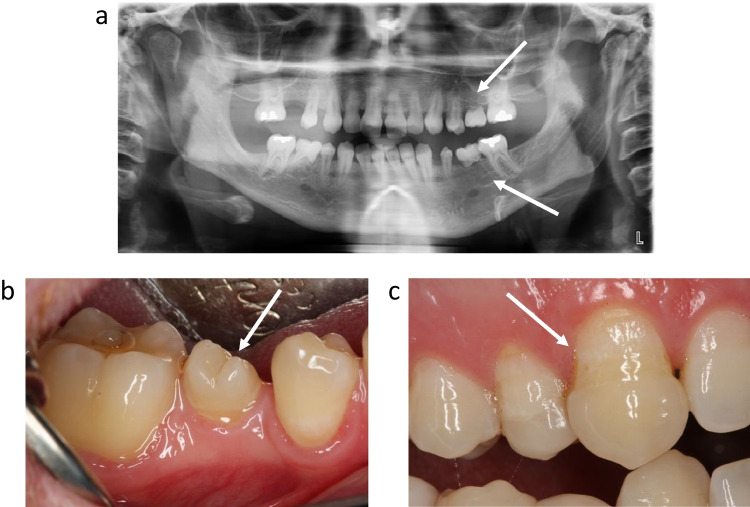
Radiograph and photos illustrating dental development disturbances recorded in survivors of childhood MB/CNS-PNET. **a** Hypodontia (missing development of a tooth) of permanent teeth. **b** Microdontia (tooth ≤ 50% of expected size). **c** Enamel hypoplasia. Abbreviations: MB medulloblastoma CNS-PNET central nervous system supratentorial primitive neuroectodermal tumor

Disturbances were only evaluated in permanent teeth. Hypodontia, missing development of a tooth, was registered if the participant was past the age of expected normal dental development [30]. Registration of DDD was done as follows (Fig. 2) [9, 15, 16]:

1. Hypodontia, when the survivor had no history of tooth extraction, or a missing tooth was obvious on radiographs (Fig. 2a)
2. Microdontia, tooth ≤ 50% of expected size (Fig. 2b)
3. Obvious enamel hypoplasia of the teeth (Fig. 2c)

### Statistical analyses

Patient characteristics were reported by descriptive statistics. Categorical variables were presented as frequencies with proportion, while continuous variables were presented as mean with standard deviation (SD) and range. Continuous variables that were not normally distributed were presented with median and interquartile range (IQR) in addition to mean (SD). The chi-square test and Fisher’s Mid-*p* test were used for comparison of proportions, as appropriate, while comparison of means was performed by an independent *t*-test. In case of non-normally distributed outcomes, comparison of median was performed using the median test. A *p* value < 0.05 was considered statistically significant. All analyses were performed using SPSS (IBM SPSS Statistics 27.0 for Windows, IBM Corp., Armonk, NY) and Stata (StataCorp. 2019. *Stata Statistical Software: Release 16*. College Station, TX: StataCorp LLC.).

## Results

### Participants

Of all 63 invited survivors, 50 (79%) consented to participate (Fig. 1). Four survivors were not able to go through an oral and radiographic examination due to severe cognitive and/or physical impairment [21]. These four individuals were excluded from the study. Ultimately, 46 (73%) survivors were included in our sub-study on oral and dental late effects.

Survivor and treatment characteristics are presented in Table 1. The mean age at treatment was 8.5 years (range 0.2–19.2 years), and the mean age at study entry was 27 years (range 5.5–51.9 years). The mean time since treatment was 18.9 years (range 3.2–40.4). In total, 40 (87%) of the 46 survivors received CSI, with a mean dose of 31.6 Gy (range 23.4–36 Gy). One patient received focal proton therapy without CSI. Five participants did not receive RT due to young age (< 3–5 years) at treatment. Thirty-eight (82.6%) of the 46 survivors received chemotherapy including alkylating agents in 18 (47.4%) and vincristine in 37 (97.4%) patients.

**Table 1:** Characteristics of 46 survivors treated for childhood MB/CNS-PNET

Gender, *n* (%)	
Female	22 (48)
Male	24 (52)
Diagnosis, *n* (%)	
MB	40 (87)
CNS-PNET	6 (13)
Age at treatment, *mean* ± *SD (years)*	8.5 ± 5.2 (range 0.2–19.2)
Age at study examination, *mean* ± *SD (years)*	27 ± 12.8 (range 5.5–51.9)
Time since treatment, *mean* ± *SD (years)*	18.9 ± 12 (range 3.2–40.4)
Treated with surgery, *n (%)*	
Yes	46 (100)
Treated with chemotherapy, *n (%)*	
Yes	38 (82.6)
Including alkylating agents	18
Including vincristine	37
No	8 (17.4)
Treated with CSI, *n (%)*	
Yes	40 (87)
Mean dose of CSI (*Gy*)	31.6 (range 23.4–36)
No	6 (13)

*MB* medulloblastoma, *CNS-PNET* central nervous system supratentorial primitive neuroectodermal tumor, *CSI* craniospinal irradiation

### Oral health parameters

#### Dental caries

Six (13%) survivors had deciduous teeth present; all of them had dmft/DMFT score 0. The mean DMFT score in all participants was 5.2 ± 6 (range 0–21). There was no significant difference in DMFT score between survivors treated at age > 5 years compared to survivors treated at age ≤ 5 years (0 vs 4, *p* = 0.139) (Table 2).

**Table 2:** Oral health parameters and dental developmental disturbances in survivors of childhood MB/CNS-PNET related to age at treatment

	Total *(n* = *46)*	≤ 5 years *(n* = *15)*	> 5 years *(n* = *31)*	*p* value
DMFT score				
*Median (IQR)*	3 (0–8)	0 (0–4)	4 (1–10)	0.139
*Mean* ± *SD (min–max)*	5.2 ± 6 (0–21)	1.7 ± 0.6 (0–6)	6.9 ± 1.2 (0–21)	
Survivors with one, or more types of DDD, *n (%)*	14 (30.4)	11	3	< 0.001
Hypodontia				
Number of survivors, *n (%)*	10 (21.7)	7	3	0.004
Number of teeth, *n*	29	24	5	
Microdontia				
Number of survivors, *n (%)*	7 (15.2)	6	1	0.002
Number of teeth, *n*	25	24	1	
Enamel hypoplasia				
Number of survivors, *n (%)*	4 (8.7)	4	No	0.004
Number of teeth, *n*	10	10	No	
Maximum mouth opening (MMO) in mm				
*Median (IQR)*	40 (33.8–45)	34 (24–37)	42 (37–46)	0.021
*Mean* ± *SD (min–max)*	38.5 ± 8.4 (16–55)	33.3 ± 2.4 (16–55)	41.1 ± 1.2 (28–51)	
MMO ≤ 35 mm, *n (%)*	16 (35)	10	6	0.002
*Mean* ± *SD (min–max)*	29.3 ± 5.6 (16–35)			
MMO > 35 mm, *n (%)*	30 (65)	5	25	
*Mean* ± *SD (min–max)*	43.5 ± 4.7 (36–55)			

*MB* medulloblastoma, *CNS-PNET* central nervous system supratentorial primitive neuroectodermal tumor, *DMFT* decayed-missing-filled-teeth, *IQR* interquartile range, *DDD* dental development disturbances

#### Oral dryness

Patient-reported and observed oral dryness outcomes are presented in Table 3. The mean SXI sum score, which indicates survivor` subjective evaluation of oral dryness, was 6.8 ± 1.8 (range 5–13). The mean CODS was 0.5 ± 1.1 (range 0–4). Eight (17.4%) survivors showed mild and one (2.2%) had moderate signs of oral dryness. The CODS features “*mirror sticking to buccal mucosa and/or tongue*” and “*frothy saliva*” were the most commonly registered. Most participants, 37 (80.4%), showed no clinical signs of oral dryness according to the CODS index. Among the nine survivors with positive oral dryness features according to the CODS index, the mean sum score of SXI was 8.4 ± 2.7 (range 5–13). Presence of oral dryness, evaluated by the sliding mirror friction test, was found in five (11%) survivors; the mean CODS score among these was 3 (range 2–4).

**Table 3:** Evaluation of oral dryness in survivors of childhood MB/CNS-PNET, *n* = 46

SXI-D score	
* Median (IQR)*	6 (6–8)
* Mean* ± *SD (min–max)*	6.8 ± 1.8 (5–13)
CODS score	
* Median (IQR)*	0 (0–0)
* Mean* ± *SD (min–max)*	0.5 ± 1.1 (0–4)
Mirror friction test, *n (%)*	
No friction	41 (89)
Friction	5 (11)
Use of medication with dry mouth as possible a side effect, *n (%)*	10 (21.7)
Use of medication with increased saliva as possible a side effect, *n (%)*	3 (6.5)

*MB* medulloblastoma, *CNS-PNET* central nervous system supratentorial primitive neuroectodermal tumor, *SXI-D* summated xerostomia inventory-Dutch version (scale range 5–15), *CODS* clinical oral dryness score index (scale range 0–10)

#### Maximum mouth opening measurement

**Fig. 3:**
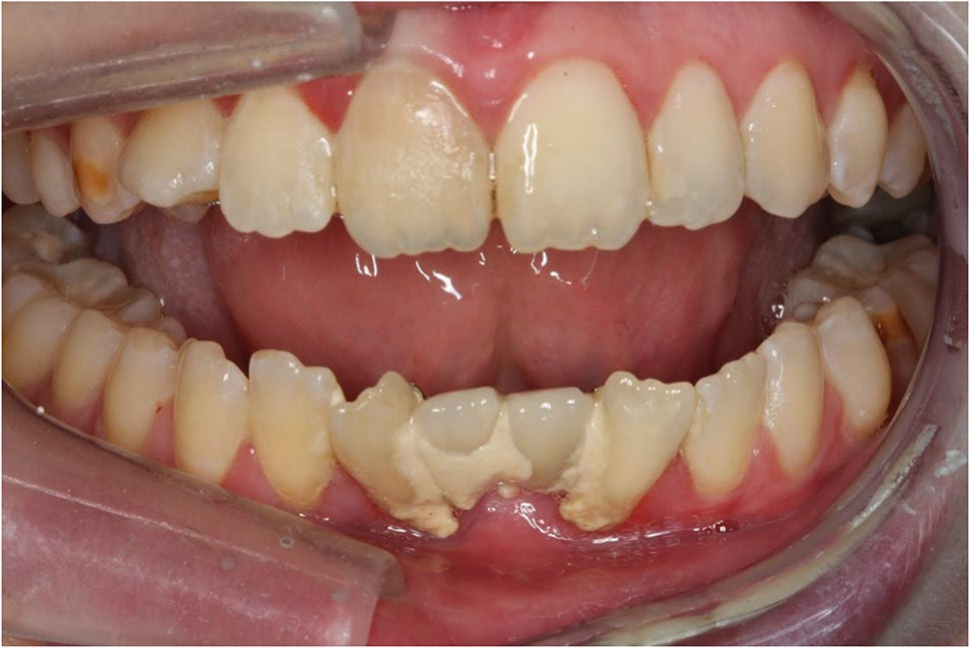
Photo illustrating maximum mouth opening of 16 mm in a 32-year-old female survivor of MB/CNS-PNET. The participant was treated at the age of 2 1/2 years. Abbreviations: MB medulloblastoma CNS-PNET central nervous system supratentorial primitive neuroectodermal tumor

The mean MMO among all participants was 38.5 ± 8.4 (range 16–55). A significantly lower MMO (*p* = 0.021) was found in survivors treated ≤ 5 years of age compared to survivors treated at an age > 5 years (Table 2). Reduced mouth opening, measured as MMO ≤ 35 mm, was found in 16 (35%) of 46 survivors. The mean score in these 16 was 29.3 ± 5.6 mm (range 16–35) (Table 2). A survivor with MMO of 16 mm is shown in Fig. 3.

#### Oral fungal infection

None of the participating survivors had clinical signs and/or a positive microbiology test of oral candidiasis.

### Dental developmental disturbances

Prevalence of DDD in survivors is listed in Table 2. Almost one-third (30.4%) of all participants had one or more DDD. Hypodontia was the most prevalent and found in 10 (21.7%) of the 46 survivors. Significant differences were found in the prevalence of total DDD, hypodontia, microdontia, and enamel hypoplasia between survivors treated ≤ 5 years of age and individuals treated > 5 years of age (Table 2).

## Discussion

This cross-sectional study is the first to investigate long-term (mean 18.9 years) oral and dental late effects in a homogenous group of childhood MB/CNS-PNET survivors. Of 46 study participants, 30.4% survivors showed DDD, and more than one-third of survivors (35%) had reduced mouth opening (≤ 35 mm) at the time of examination. Both parameters were more prevalent in survivors treated before the age of 5.

A reduced mouth opening may affect oral function, nutritional status, oral hygiene, dental care, and health-related quality of life [7, 31]. RT-induced trismus, MMO ≤ 35 mm, in adult head and neck cancer (HNC) survivors is well known [28, 31, 32], while the reported prevalence of reduced mouth opening in childhood brain cancer survivors may vary [7, 17, 33–35]. Cetnier and coworkers found no limited mouth opening in survivors of different childhood cancers [17], while the prevalence in survivors of childhood nasopharyngeal cancer has been reported to range from 7 to 27%, with a higher risk in individuals receiving RT doses above 50 Gy [7, 33–35]. As RT-induced craniofacial growth disturbance may occur in survivors of childhood cancers [7, 10, 20, 36], it is not unlikely that this may affect the range of mouth opening. Additionally, trismus may be associated with effects of RT to the temporomandibular joint, mandible, or muscles of mastication [7].

The criteria of reduced MMO used in the present study is validated for head and neck cancer patients at the age > 18 years [28]. It should be noted that our study sample included survivors younger than 18 years at the time of study examination. However, previous studies assessing trismus in childhood nasopharyngeal cancer survivors have included those below the age of 18, the youngest being 7 years of age [33–35].

The prevalence of hypodontia, the absence of a tooth development, has been reported to be 4.5–6.5% in the Norwegian population (third molar excluded) [37, 38]. In our study, a higher prevalence of hypodontia (21.7%) was found, but this corresponds well with reported hypodontia frequencies in other childhood cancer populations (5 to 31%) [9, 12, 16–19, 39, 40]. However, it should be noted that cancer diagnosis, treatment modality, and criteria of hypodontia registration differ among the studies [9, 12, 16–19, 39, 40].

Hypodontia was the most common DDD in our study, in 21.7% of survivors, microdontia the second most common in 15.2%, and enamel hypoplasia the less common in 8.7%. In contrast, the Norwegian study by Wilberg and coworkers (2016) on 111 survivors of childhood leukemia, treated with chemotherapy at an early age, hypodontia was found in 5%, microdontia in 28%, and enamel hypoplasia in 46% [9]. The higher prevalence of hypodontia in our study population compared to childhood leukemia survivors treated with chemotherapy [9] may be due to treatment modalities combining CSI with chemotherapy [40] in MB/CNS-PNET patients.

Since most survivors in our study were treated with the combination of CSI and chemotherapy, it is difficult to evaluate the independent impact of different treatment modalities on dental development [6]. Several previous studies reported that vincristine and alkylating substances such as cyclophosphamide may be associated with DDD in childhood cancer survivors [6, 11, 41, 42]. In contrast, in a recent published study no association between DDD and specific type of chemotherapy agent was reported [40]. Even though brain tumor patients are not directly irradiated towards the jaws and teeth, scattering to these surrounding tissues may occur [43]. Sonis and coworkers (1990) compared survivors of childhood acute lymphoblastic leukemia (ALL) treated with chemotherapy alone with ALL patients treated with chemotherapy in combination with either 18 Gy or 24 Gy cranial irradiation. They found an association between severity of DDD and treatment including cranial irradiation, especially in patients receiving 24 Gy compared to 18 Gy [10]. The mean CSI dose was higher in our study population (31.6 Gy, range 23.4–36 Gy), which may indicate even greater risk of DDD.

In concurrence with previous studies [8–10, 16, 18, 41], we found the risk of DDD to be significantly associated with cancer treatment performed before the age of 5 [8–10, 16, 18, 41].

Participants’ self-evaluation of oral dryness (mean SXI score 6.8 ± 1.8) indicated that the overall group of survivors did not have a problem with dry mouth. However, the range of SXI score (5–13) among participants showed a wide variation within the group of MB/CNS-PNET survivors. It should be noted that neurocognitive function in long-term survivors of MB/CNS-PNET may also vary [21]. The ability to reflect on their subjective feeling of oral dryness may thus be compromised. Most of the survivors, 80.4%, had no clinical signs of oral dryness. The rest (19.6%) showed 2–4 clinical oral dryness signs where “*mirror sticking to buccal mucosa and/or tongue*” and “*frothy saliva*” were the most frequent signs. In comparison, a Norwegian study using the same methods on survivors of adult HNC reported a mean SXI score of 11.9 ± 2.5, and 45% of 29 participants had clinical signs of severe oral dryness (CODS score > 6) [44].

There are conflicting reports on oral dryness in survivors of different childhood cancers [7, 9, 34, 45]. Wilberg and coworkers (2016) reported xerostomia in 23% of 111 long-term survivors of childhood leukemia (mean age of 29.1 years at examination) [9], while as many as 88% of 17 pediatric survivors of nasopharyngeal cancer treated with chemo-radiation reported xerostomia in the study of Sahai and coworkers (2016) [45]. In contrast, Küpeli and coworkers reported xerostomia in only three (1.2%) of 84 survivors of childhood nasopharyngeal carcinoma [34]. Reported xerostomia in survivors of childhood cancer may vary due to different factors like how xerostomia was addressed, type of study population and treatment modality, age at treatment, and time since treatment [9, 34, 45].

Several studies have examined the risk of dental caries in childhood cancer survivors [9, 12, 13, 17]. Cetiner and coworkers (2019) found no significant difference in mean dmft/DMFT score between survivors of different childhood cancers and healthy controls [17]. In contrast, other studies have shown an increased risk of dental caries in childhood cancer survivors [12, 13]. In the Danish study by Wogelius and coworkers (2008), an increased risk of dental caries was found in survivors treated after the age of 5 years, compared to survivors treated before the age of 5 [13]. Similar results were reported by Wilberg and coworkers (2016) in survivors of childhood leukemia, where treatment after the age of 5 years was associated with a significantly higher mean DMFT score compared to survivors younger than 5 years at treatment [9]. Our findings on DMFT in MB/CNS-PNET survivors are in discordance with these studies, as we found no significant difference in DMFT score between participants treated ≤ 5 years or after the age of 5 years. Why there are no differences between the groups in our study is not clear. One theory is that childhood survivors of malign brain tumors often have multiple severe sequela after treatment [3], and therefor oral hygiene are more closely monitored by a parent/guardian and the health system.

The relatively large homogenous study population of long-term survivors of childhood malignant brain tumors and the long median time since treatment are strengths of this study. A possible limitation is the survivors who were not able to participate due to severe late effects. Additionally, the lack of a matched control group is a limitation. Furthermore, oral and dental late effects may change over time during cancer treatment, and we did not have a baseline oral and dental clinical and/or radiographical examination of participants.

This study revealed oral and dental late effects including a reduced mouth opening and DDD in long-term survivors of childhood MB/CNS- PNET. The risk of developing a reduced mouth opening and DDD seems to be related to treatment at a young age ≤ 5 years. Our findings emphasize the importance of patient information and a close and careful follow-up of these patients by health personnel with knowledge on monitoring oral and dental health years after treatment.

## Data Availability

Can be available from the corresponding author if requested.
